# Interspinous bursitis is common in polymyalgia rheumatica, but is not associated with spinal pain

**DOI:** 10.1186/s13075-014-0492-2

**Published:** 2014-12-01

**Authors:** Dario Camellino, Francesco Paparo, Silvia Morbelli, Maurizio Cutolo, Gianmario Sambuceti, Marco A Cimmino

**Affiliations:** Research Laboratory and Academic Division of Clinical Rheumatology, Department of Internal Medicine (Di.M.I.), University of Genova, Viale Benedetto XV 6, Genova, 16132 Italy; Division of Radiology, E.O. Ospedali Galliera, Mura delle Cappuccine 14, Genova, 16128 Italy; Nuclear Medicine Unit, Department of Health Sciences, University of Genova, Via A. Pastore 1, Genova, 16132 Italy

## Abstract

**Introduction:**

Polymyalgia rheumatica (PMR) is a common inflammatory disease in older people characterized by shoulder and/or pelvic girdle, and cervical and, occasionally, lumbar pain. Interspinous bursitis has been suggested as a potential cause of spinal symptoms. We evaluated, by 18 F-fluorodeoxyglucose (FDG) positron emission tomography integrated with computed tomography (PET/CT), the vertebral structures involved in PMR in a cohort of consecutive, untreated patients.

**Methods:**

Sixty-five consecutive patients with PMR were studied. After a standardized physical examination, which included evaluation of pain and tenderness in the vertebral column, they underwent FDG-PET/CT. Sites of increased uptake and their correlation with spontaneous and provoked pain were recorded. For comparison, FDG-PET/CT was performed also in 65 age- and sex-matched controls and in 10 rheumatoid arthritis (RA) patients.

**Results:**

The most frequent site of spontaneous and provoked pain was the cervical portion. FDG uptake was more frequent in the lumbar portion than at any other location, and in the cervical rather than in the thoracic portion (*P* <0.0001). No correlation was found between uptake and spontaneous or provoked pain. There was an association between presence of cervical and lumbar bursitis (r = 0.34, *P* = 0.007). None of the control patients and one out of ten RA patients showed interspinous bursitis.

**Conclusions:**

Interspinous bursitis is a frequent finding in the lumbar spine of patients with PMR. However, it is not associated with clinical symptoms and can hardly explain the spinal pain reported by the patients. Cervical pain is more frequent than lumbar pain in PMR patients and may be caused by shoulder girdle involvement.

## Introduction

Polymyalgia rheumatica (PMR) is a common inflammatory disease of older people characterized by shoulder and/or pelvic girdle pain and cervical and lumbar tenderness [1]. Its cause is still unknown and even the anatomical site of inflammatory damage is unclear, for synovitis, vasculitis, and bursitis have been advocated. These different mechanisms could be simultaneously at work in the pathogenesis of PMR, as suggested by anecdotal observations [2]. Interspinous bursitis has been suggested as a potential cause of the symptoms in the cervical and lumbar segments in two studies performed with magnetic resonance imaging (MRI) [3,4] and in one small series investigated with ultrasonography [5]. Interspinous bursae are usually found in narrow slits at the inferior part of the upper spinous process [6]. They are lined by synovial cells, and are more frequent in older subjects, especially in the lower cervical and lumbar spaces [7].

Interspinous bursae were not included in an extensive treatise of human bursae published in 1788 [8], but were first described by Mayer in 1825 [9]. Interspinous bursitis is seen in several rheumatic diseases, including rheumatoid arthritis (RA), juvenile idiopathic arthritis and calcium pyrophosphate deposition disease [10,11]. It has also been described in Baastrup’s disease, a condition characterized by close proximity or contact of adjacent spinous processes with resultant enlargement, flattening, and reactive sclerosis of the pertinent adjacent interspinous surfaces [12].

In rheumatology, 18 F-Fluorodeoxyglucose (FDG) positron emission tomography integrated with computed tomography (PET/CT) is used mainly to demonstrate large-vessel vasculitis. Characteristic uptake patterns are also seen in PMR, where bursal and joint involvement is highlighted in shoulders, hips, column, and ischium [13]. Two different studies have shown abnormal uptake in the spine of PMR patients at the level of the spinous processes [14,15], without a precise anatomical localization of the inflammation. The aim of our study was to evaluate by FDG-PET/CT the spine structures involved in PMR and the correlation between interspinous bursitis and clinical findings in a cohort of consecutive, untreated patients.

## Methods

### Patients

Sixty-five consecutive new patients (44 women and 21 men, median age 73 years, range 50 to 87 years), fulfilling Bird’s criteria for PMR at enrolment [16] were studied. Retrospective evaluation of their charts showed that they also fulfilled the American College of Rheumatology (ACR)/European League Against Rheumatism (EULAR) criteria for PMR published in 2012 [17]. None of the patients had clinical or histological evidence of giant cell arteritis or had been previously treated with steroids. We defined as ‘pure PMR’ all the patients since none of them fulfilled ACR criteria for temporal arteritis. The imaging protocol was approved by the ethics committee of the University of Genova and the patients signed an informed consent document. A standardized physical examination, which included pain in the girdles elicited by passive and active movements, degree of elevation of upper arms, and pain and tenderness of the vertebral column was performed. Pain presence was recorded in a dichotomous manner (present or absent). Disease duration, duration of morning stiffness, and presence of fever, weight loss, and peripheral arthritis were recorded. During a follow-up that lasted for a median of 22 months (range 5 to 59 months) none of the patients developed RA or a different rheumatic condition. For comparison, 65 age- and sex-matched control patients without inflammatory disorders, who underwent PET/CT to rule out malignancy, and 10 patients with RA (7 women, mean age 65.2 ± 14.8 years), were evaluated.

### Imaging acquisition and analysis

Patients underwent simultaneous FDG-PET and CT imaging from the skull base to the knee using an integrated PET/CT scanner (Hirez; Siemens Medical Solutions, Knoxville TN, USA), after injection of 4.8 to 5.2 megabecquerel (MBq) of F18-FDG per kilogram body weight. FDG uptake was scored semi-quantitatively in agreement by a rheumatologist experienced in FDG-PET uptake in inflammatory disorders (D.C.) and by a radiologist expert in musculoskeletal diseases (F.P.). Scores were attributed according to liver uptake as 0 = no uptake present, 1 = lower than liver uptake, 2 = similar to liver uptake, 3 = higher than liver uptake, as proposed by Walter *et al*. [18]. These values were further subdivided into ‘positive’ (scores 2 and 3) and ‘negative’ (scores 0 and 1). Sites of pathological uptake in the column and the portion concerned were recorded. In addition, to look for alternative explanations for cervical pain, increased uptake of the carotid arteries, suggestive of large vessel vasculitis, was also evaluated. Possible co-morbidity with Baastrup’s disease was considered for on the CT images. C-reactive protein (CRP) and erythrocyte sedimentation rate (ESR) were assessed as inflammatory markers.

### Statistical analysis

Means were compared by the Student’s *t* test or by one way analysis of variance if their distribution was normal and by the Kruskall-Wallis test when it was non-parametrical. Frequencies were compared by the chi-square test. Correlations were calculated by the Pearson’s method. All the calculations were performed using Medcalc™ version 12.3 (MedCalc Software, Mariakerke, Belgium) as statistical software.

## Results

**Figure 1:**
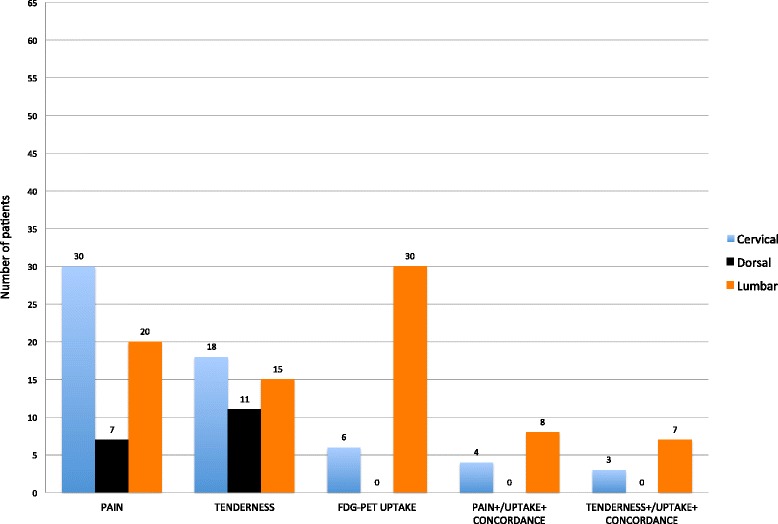
**Location of spontaneous pain, tenderness, FDG uptake, and their concordance, in the different areas of the spine of polymyalgia rheumatica patients.** FDG, 18 F-fluorodeoxyglucose.

**Figure 2:**
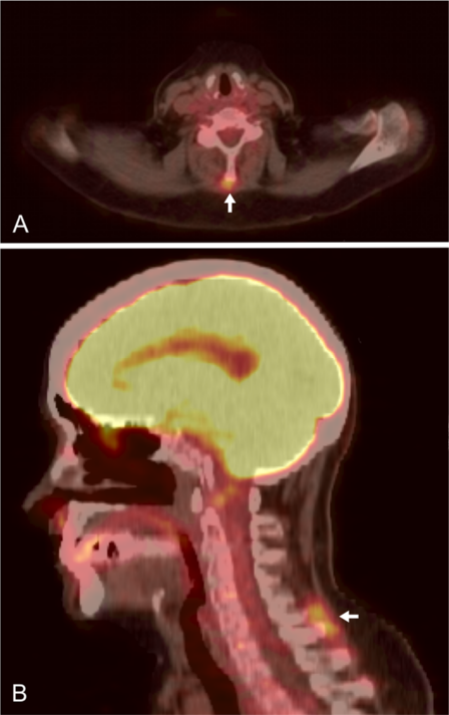
**FDG uptake in the cervical interspinous spaces of a patient with polymyalgia rheumatica. (A)** Axial PET/CT section at the level of the 5^th^ cervical vertebra; **(B)** sagittal PET/CT image showing bursitis (arrows) between 5^th^ and 6^th^ cervical vertebra. FDG, 18 F-fluorodeoxyglucose; PET/CT, positron emission tomography integrated with computed tomography.

**Figure 3:**
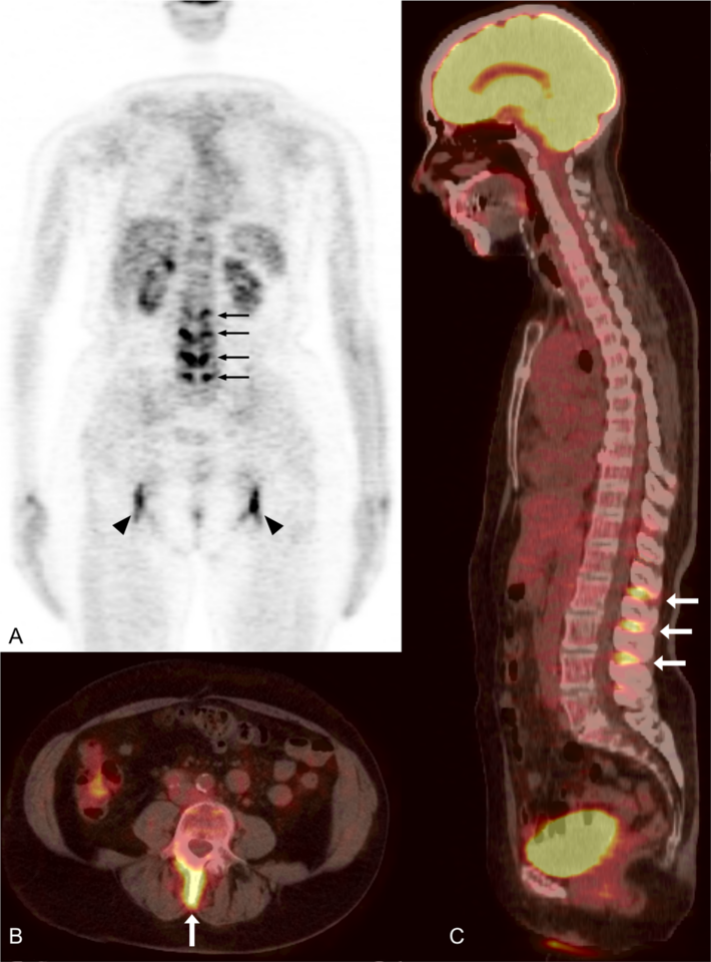
**FDG uptake in the lumbar interspinous spaces of a patient with polymyalgia rheumatica. (A)** Coronal PET image; **(B)** axial PET/CT section at the level of the 3^rd^ lumbar vertebra; **(C)** sagittal PET/CT image showing interspinous bursitis (arrows) between 1^st^ and 2^nd^, 2^nd^ and 3^rd^, 3^rd^ and 4^th^ lumbar vertebrae. FDG, 18 F-fluorodeoxyglucose; PET/CT, positron emission tomography integrated with computed tomography.

**Figure 4:**
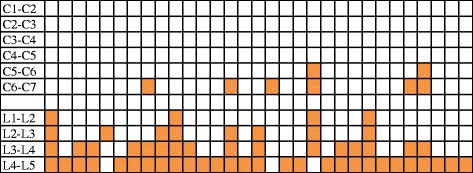
**The localization of bursitis in the interspinous spaces is indicated by the colored box.** Each column represents a patient with bursitis.

Median disease duration was 68 days (range 4 to 486 days), median morning stiffness was 60 minutes (range 15 to 360 minutes), median CRP was 36 mg/L (range 2 to 149 mg/L), and median ESR was 59 mm/h (range 10 to 120 mm/h). Clinically, the most frequent site of spontaneous and provoked pain was the cervical spine (Figure 1). FDG uptake suggestive of interspinous bursitis was seen only in the cervical and lumbar spine. It occurred in six patients (9%) at the cervical level (Figure 2) and in 30 (46%) at the lumbar level (Figure 3) (*P* <0.0001 for comparison between lumbar spine and the other locations, and between cervical and thoracic spine). No uptake was present in the vertebral column of control patients; in comparison with them, PMR patients showed more frequently cervical (*P* = 0.04) and lumbar (*P* = 0.001) bursitis. One patient with RA showed interspinous bursitis between lumbar vertebrae 3 to 4. Thirty-one out of 65 (47.7%) PMR patients showed interspinous bursitis in comparison with one out of ten (10%) RA patients, *(P* = 0.037). PMR patients, had uptake scores higher in the lumbar than in the cervical spine (1.3 ± 1.2 vs. 0.3 ± 0.8, *P* <0.0001). Uptake was more frequently observed at cervical vertebrae 3 to 6 and lumbar vertebrae 4 to 5. The number and location of the involved bursae in the individual patients is shown in Figure 4. No association was found between FDG uptake and spontaneous or provoked pain. Cervical and lumbar bursitis correlated (r = 0.34, *P* = 0.007) and often coexisted in the same patient. No correlation was found between uptake in the vertebral column and age, sex, disease duration, morning stiffness, CRP and ESR, or peripheral arthritis. Baastrup’s syndrome was not identified in any of these patients or in controls. Carotid artery vasculitis was seen in only three out of 65 (4.6%) patients, but was not associated with cervical pain. Of these three patients with vasculitis, one had pain but no tenderness of the cervical column, another had tenderness but no pain, and the last one had neither symptom.

## Discussion

Interspinous bursitis is a frequent finding in the lumbar column of patients with PMR, less so in the cervical column, but has not been demonstrated in their thoracic column. It was absent from controls without rheumatic diseases and occurred in one out of ten patients with RA. The formation of interspinous bursae is probably promoted by enhanced mobility due to damaged supraspinous and interspinous ligament with instability of the adjacent spinous processes [6]. These events can occur in degenerative conditions, such as Baastrup’s disease [12]. In our study, both PMR and control patients may have had mild asymptomatic spine osteoarthritis, but examination of the CT images excluded the presence of Baastrup’s syndrome as a cause of interspinous bursitis.

The reason why only the lumbar and, to a lesser degree, the cervical column of PMR patients were interested by bursitis is not known. A possible explanation is that these are the more flexible segments of the vertebral column, and hence more frequently the target of microtrauma that may elicit local inflammation. The lumbar spine, which was more heavily affected, also bears more weight. Mechanical overload may represent the triggering event of bursitis at this location. These two locations are those that PMR patients more often describe as painful (Figure 1). However, in our series, FDG uptake was not associated with severity or appearance of clinical symptoms. As a result, we doubt that interspinous bursitis can explain the spinal pain reported by PMR patients. Alternatively, the absence of correlation between bursal uptake and symptoms could be due to a low sensitivity of PET/CT in detecting spinal bursitis.

PMR has been interpreted as a disease of the extra-articular synovial membrane, including bursae, such as the subacromial and trochanteric ones [19], and synovial sheaths [20]. Our findings support the view that interspinous bursae can also contribute to the inflammatory load of PMR patients, although these lesions seem to be unrelated to clinical symptoms. Cervical pain, which is clinically more frequent than lumbar pain, is probably unrelated to bursitis, but may be elicited, among other causes, by involvement of the adjacent shoulder girdle. The hypothesis that carotid arteries vasculitis could induce pain in the adjacent spine structures is contradicted by the lack of association between this lesion and cervical pain.

Only one of the control patients had interspinous bursitis, a fact which suggests a high specificity of this finding for PMR. In contrast, Salvarani *et al*. [3] found interspinous bursitis with MRI in patients with fibromyalgia or mild osteoarthritis. This difference could be due to the fact that the controls of our first group were not affected by rheumatic conditions, as demonstrated from the review of their clinical charts. Alternatively, PET/CT may have a lower sensitivity compared with post-contrast MRI, the imaging technique used in the other studies. In fact, Salvarani *et al*. found a prevalence of cervical bursitis of 100% [3] and of lumbar bursitis of 90% [4]. A study comparing MRI and PET/CT for the evaluation of interspinous bursitis could be of interest. Bursal uptake evaluation using standardized uptake value (SUV) instead of the used semi-quantitative score could have enhanced the sensitivity of PET/CT. However, the former method cannot be used in controls and unaffected patients because it implies the positioning of a region of interest (ROI) on the bursa, which is only virtual in these subjects. The major strength of our study is the examination of a large sample of untreated patients with pure PMR, in which location of tenderness of the spinous processes was specifically searched. An additional strength is the presence of a control group with RA, albeit small, in which the differential diagnostic value of interspinous bursitis could be evaluated. Although not specific for PMR, interspinous bursitis was significantly more frequent than in RA.

## Conclusions

Interspinous bursitis revealed by PET/CT was frequent in PMR, especially at the lumbar level. Nonetheless, this finding was not associated with pain, which was more frequently reported by the patients at the cervical level. As a result, inflammation at the interspinous bursae cannot be considered the cause of clinical symptoms in PMR patients.

## Abbreviations

ACR: American College of Rheumatology
CRP: C-reactive protein
ESR: erythrocyte sedimentation rate
EULAR: European League Against Rheumatism
FDG: 18 F-fluorodeoxyglucose
MBq: megabecquerel
MRI: magnetic resonance imaging
PET/CT: positron emission tomography integrated with computed tomography
PMR: polymyalgia rheumatica
RA: rheumatoid arthritis
ROI: region of interest
SUV: standardized uptake value
